# Impact of Breast Size on Dosimetric Indices in Proton Versus X-ray Radiotherapy for Breast Cancer

**DOI:** 10.3390/jpm11040282

**Published:** 2021-04-08

**Authors:** Lisa Cunningham, Scott Penfold, Eileen Giles, Hien Le, Michala Short

**Affiliations:** 1 Department of Radiation Oncology, Royal Adelaide Hospital, Adelaide 5000, Australia; Lisa.Cunningham@sa.gov.au (L.C.); Scott.Penfold@sa.gov.au (S.P.); hien.le@sa.gov.au (H.L.); 2 Cancer Research Institute, University of South Australia, Adelaide 5001, Australia; eileen.giles@unisa.edu.au

**Keywords:** breast cancer, breast size, intensity-modulated radiotherapy, precision medicine, proton therapy, radiotherapy planning

## Abstract

Deep inspiration breath hold (DIBH) radiotherapy is a technique used to manage early stage left-sided breast cancer. This study compared dosimetric indices of patient-specific X-ray versus proton therapy DIBH plans to explore differences in target coverage, radiation doses to organs at risk, and the impact of breast size. Radiotherapy plans of sixteen breast cancer patients previously treated with DIBH radiotherapy were re-planned with hybrid inverse-planned intensity modulated X-ray radiotherapy (h-IMRT) and intensity modulated proton therapy (IMPT). The total prescribed dose was 40.05 Gy in 15 fractions for all cases. Comparisons between the clinical, h-IMRT, and IMPT evaluated doses to target volumes, organs at risk, and correlations between doses and breast size. Although no differences were observed in target volume coverage between techniques, the h-IMRT and IMPT were able to produce more even dose distributions and IMPT delivered significantly less dose to all organs at risk than both X-ray techniques. A moderate negative correlation was observed between breast size and dose to the target in X-ray techniques, but not IMPT. Both h-IMRT and IMPT produced plans with more homogeneous dose distribution than forward-planned IMRT and IMPT achieved significantly lower doses to organs at risk compared to X-ray techniques.

## 1. Introduction

Breast cancer is the most commonly diagnosed cancer among women. Based on global incidence data, 2.1 million new cases of breast cancer were diagnosed in 2018 [1]. In countries such as Australia, mean age at diagnosis is 61.4 years, with treatment involving a combination of surgery, chemotherapy, radiotherapy, and hormone therapy. Treatments are effective—patients can expect a 90% rate of survival five years after diagnosis [2]. With such favorable survival outcomes in a relatively young group of women, it is important to consider patients’ long-term quality of life, as treatment should not only cure the disease, but also do so with minimal side effects.

Treatment of left-sided breast cancer with radiotherapy (RT) carries risk of late side effects to the heart and lungs. The importance of minimizing dose to these organs at risk (OAR) is well recognized, as lower doses reduce patients’ risk of ischemic heart disease, lung fibrosis, and second primary lung cancer. Deep inspiration breath hold (DIBH) techniques enable reduction of dose to these healthy structures, decreasing the risk of side effects. Hybrid inverse-planned intensity modulated X-ray radiotherapy (h-IMRT) DIBH treatment is the current standard of care for early stage left-sided breast cancer patients at our institution in Australia. In this approach, part of the dose is delivered by two open tangential beams, and intensity modulated beams are calculated by an inverse planning algorithm to deliver the remaining dose. This technique, originally developed by Mayo et al. [3] allows for efficient and consistent treatment planning, however, an alternative approach using proton therapy offers potential to further reduce doses to healthy tissue.

Investigation into proton therapy for breast cancer began in 2002, with the first retrospective breast proton planning studies conducted in Switzerland and Sweden [4,5,6]. These studies found that proton therapy delivered comparable target coverage with lower doses to healthy tissues, including lungs and heart, when compared to X-ray techniques. Recent prospective studies confirm the early work and feasibility of using proton therapy for whole breast (WB) and chest wall radiotherapy, with promising patient outcomes [7,8].

Preventing long-term side effects to heart and lungs is the main predicted benefit of proton therapy for WB and chest wall irradiation, although currently there are no published data on long-term follow-up of breast patients after proton treatment. Bradley et al. reported follow-up at median 20 months [7], however, this follow-up period is not long enough to report late cardiac events, which are expected between 5 and 20 years post-treatment [9]. The first randomized control trial for breast proton versus X-ray therapy was initiated by the University of Pennsylvania in 2016. The Radiotherapy Comparative Effectiveness (RadComp) Study is a pragmatic, randomized clinical trial of protons versus X-rays for patients with stage I-III non-metastatic breast cancer requiring treatment of the breast or chest wall with inclusion of regional lymphatic nodes and internal mammary nodes. The primary outcome measure of the trial is to compare the effectiveness of proton and X-ray therapy in reducing major cardiovascular events up to 10 years post-treatment. The trial is currently in-progress and aims to recruit 1278 patients, with final data collection estimated August 2022 [10]. No results from the trial have been released to date.

The focus of our research was to update international findings by providing a comparison of the latest in breast X-ray and proton planning techniques. Breast radiotherapy using deep-inspiration breath hold and hypo-fractionated schedules are now standard of care for patients with invasive breast cancer [11,12]. In addition, most aforementioned proton therapy studies used passive scattering techniques with free-breathing, whereas pencil-beam scanning technology is now being more widely adopted in clinical practice [13]. The primary aim of this study was to compare DIBH X-ray and proton therapy plans in women with early stage left-sided breast cancer to explore differences, if any, in target coverage, doses to organs at risk, and impact of breast size on target coverage and doses to organs at risk.

## 2. Materials and Methods

### 2.1. Ethical Considerations and Data Sources

Ethics approval for this study was obtained from the Human Research Ethics Committees of both the Royal Adelaide Hospital (protocol number HREC/15/RAH/127) and the University of South Australia (Application ID 0000035113). This study was a retrospective dosimetric comparison of three radiotherapy planning techniques: (1) hybrid forward-planned IMRT (fp-IMRT); (2) hybrid inverse-planned IMRT (h-IMRT); and (3) intensity modulated proton therapy.

Inclusion criteria consisted of females over 18 years of age, diagnosed with early stage left breast cancer (T1/T2, N0/M1mic, M0) who received radiotherapy using a DIBH technique to a total dose of 40.05 Gy in 15 fractions to the whole breast. Patients were excluded if they had a pacemaker or implantable cardioverter defibrillator, breast expanders or prosthesis, mastectomy, treated with bolus or had previous radiotherapy. All patients were positioned supine with both arms up and lying on a raised breast board according to standard department protocol. All radiotherapy plans were created in the Pinnacle Treatment Planning System (Pinnacle 9.10, Philips Medical Systems 2014).

Records were searched from April 2016, when the DIBH technique was adopted at this institution. In total 16 patients met the inclusion criteria. Patients’ Computed Tomography (CT) images along with their radiotherapy plans were de-identified and recoded with a unique study ID by one of the co-authors to preserve confidentiality, anonymity, and to ensure a blinded planning process by the lead author. Original clinical plans were created using a hybrid fp-IMRT and were copied with regions of interest (ROIs) defined in Appendix A Table A1 based on local and international guidelines [14,15,16,17].

### 2.2. Planning Procedure

The volume of tissue requiring treatment was defined as the Clinical Target Volume (CTV) and comprised all apparent glandular breast tissue plus lumpectomy site CTV as visible on CT, according to established international guidelines for breast radiotherapy [14]. An experienced radiation oncologist checked all ROIs for accuracy. Once this was confirmed, the beam angles and energies of the fp-IMRT plans were recorded and plans were copied twice with beams, points, and prescriptions deleted. The copied plans were then renamed to h-IMRT and IMPT and new plans were generated to a prescribed total dose of 40.05 Gy (40.05 Gy Relative Biological Effectiveness (RBE) for the proton plans, RBE value was 1.1) in 15 fractions [12]. The planning goals for both modalities (Appendix A Table A2) were to achieve a consistent dose to the target volume, greater than 98% coverage of the PTV with 95% of the prescribed dose, maximum effective dose of less than 107% of the prescribed dose [17] and organ at risk doses within current Australian guidelines [15].

The h-IMRT plans consisted of four to six tangent beams where each h-IMRT plan had 2–4 open tangents and 2 IMRT tangents with the same gantry angles and beam energy to match the original plan and thus enable a true dosimetric comparison. Seventy percent of the dose was delivered by the open fields and 30% by the IMRT fields with dose prescribed to a point. These were inversely optimized using direct machine parameter optimization, as described in similar studies [18,19]. The open fields shielded the PTV with a 5 mm margin, and had 3 cm flash to provide adequate coverage to the anterior breast tissue and allow for breathing motion.

The proton plans were generated using a pencil beam scanning (PBS) technique. Plans had a single en-face beam; the planner selected the angle on a case-by-case basis depending on patient anatomy. One field was deemed sufficient to treat the volume as applying extra fields would unnecessarily increase both healthy tissue irradiation and treatment time [4]. A beam-specific PTV was generated, with an expansion of 5 mm in superior, inferior, left and right directions and 1 mm distally to the CTV. This 1 mm expansion in the beam direction was to account for 3% range uncertainty, which corresponds to be between 0.3 and 1 mm in tissue for typical beam ranges encountered in en-face breast delivery. Since the treatment planning system in use did not allow for percentage range uncertainties when generating beam specific volumes, fixed dimensions were required. The beam-specific PTV was modified to exclude the left lung and trimmed 5 mm from the skin. The lateral margin for spot placement was 4 mm uniformly around the PTV, with spot placement calculated automatically by the planning system. In depth, 80% overlap was allowed between spot layers. Proton energies of 85–250 MeV were available, and a range shifter of 6.7 cm water equivalent thickness was used to ensure superficial target coverage. A 5 cm air gap was allowed between the snout and the patient.

Single field uniform dose to the PTV was utilized for beam spot weight optimization. While robust optimization to a CTV is becoming more common in PBS treatment planning, single field uniform dose to the PTV is justified for this study due to use of only one beam, the relatively uniform breast tissue through which the beam is passing and the relatively low tissue gradients encountered across potential inter- and intra-fraction motion.

Final dose computation for both X-ray and proton plans was achieved using a 0.2 × 0.2 × 0.2 cm dose grid, with an adaptive convolve dose algorithm for X-ray plans and pencil beam algorithm for proton plans. Additional beam parameters are shown in Appendix A Table A3. Face validity of completed plans was provided by a medical physicist and radiation oncologist.

### 2.3. Statistical Analysis

All variables were initially analyzed using descriptive statistics, with the calculation of the mean and standard deviation for continuous variables and medians and interquartile ranges for planning parameters that did not satisfy normality assumptions. To ensure highest level of planning reliability, all planning was performed by a single observer and a blinded investigation of intra-observer reliability was undertaken to determine the degree of planning consistency. Two duplicate patients were added at the initial de-identification step meaning that on a separate occasion, the planner created an additional pair of plans for two patients. Intra-observer reliability was determined by converting DVH data into an area under the curve (AUC), which allowed the overall DVH curve for each region of interest to be captured as a single, standardized measure. The AUC data were then compared across the two time-points using intra-class correlation coefficients (ICC) along with 95% confidence intervals, with ICC values less than 0.5 indicating poor, 0.5–0.75 moderate, 0.75–0.9 good, and >0.90 excellent reliability [20].

After reliability testing, comparisons across the three sets of plan data were performed using the non-parametric Friedman two-way ANOVA and statistically significant differences were followed up with a or Wilcoxon signed rank test.

Associations between CTV volume (proxy for breast size) and dosimetric results were investigated using non-parametric Kendall’s tau-B correlation tests. All data analysis was completed using the SPSS software (SPSS version 25, IBM Corp. 2017) with a level of statistical significance set at *p* ≤ 0.05 for all tests, except the follow-up pair-wise comparisons which had a Bonferroni correction applied to adjust for multiple comparisons (*n* = 3) with a corresponding adjustment to level of significance to *p*-values ≤ 0.017.

## 3. Results

### 3.1. Patient Characteristics

In total 59 potential patients were identified, with 39 excluded after eligibility testing providing 20 eligible patients. Of these, four were excluded; two due to corrupt planning data files and two due to unsuitable target volumes, giving a final sample of 16 patients. All 16 patients received breast-conserving surgery and were treated with post-operative radiotherapy to the whole breast using a DIBH technique. Patients’ mean age at diagnosis was 57.1 years (SD = 11). Mean CTV volume was 808 cm^3^ with minimum to maximum volumes ranging from 219–1562 cm^3^. Patient characteristics are shown in Table 1.

### 3.2. Reliability Study

The blinded intra-observer reliability study showed ICC of 0.898 (*p* < 0.001, 95% confidence interval 0.782–0.955) which indicated good test-retest reliability. Given that CTV and PTV plan parameters are likely to be highly consistent across planning attempts as these were the primary planning end-points, the ICC were repeated with CTV and PTV data excluded in order to minimize this potential bias. The resulting ICC remained high at 0.884 (*p* < 0.001, 95% confidence interval 0.707–0.958) and therefore the planning data generated in this study was deemed to have good reliability.

### 3.3. Dosimetric Indices

**Figure 1:**
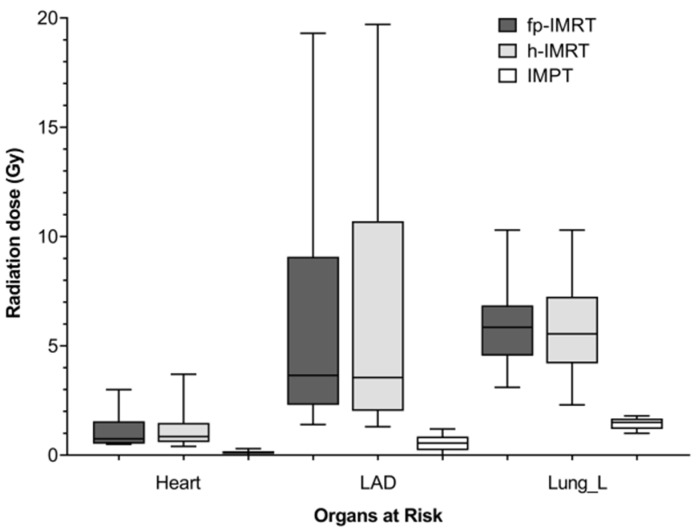
Boxplots showing mean doses received by the heart, LAD, and left lung grouped by technique. Boxes show interquartile range, line within box indicates median and whiskers show minimum and maximum values. All *n* = 16. Abbreviations: fp-IMRT—forward planned intensity-modulated radiation therapy; h-IMRT—hybrid intensity-modulated radiation therapy; IMPT—intensity modulated proton therapy; LAD—left anterior descending artery.

The target volume coverage when assessed using PTV metrics of D98% and V38.05 Gy was not found to be significantly different across the three techniques, however significant differences were observed in the remaining target volume and all organ at risk plan parameters as shown in Table 2 and Table 3, respectively. Figure 1 shows a comparison of mean doses to the heart, LAD, and left lung across techniques. As mean doses to the right lung and right breast were near zero these are not included in Figure 1. Pairwise comparisons showed that the h-IMRT and IMPT resulted in a significantly lower homogeneity index and significantly lower CTV and PTV mean and maximum doses compared to the fp-IMRT plan, and that the h-IMRT plan resulted in significantly lower near minimums compared to the IMPT plan. For the organs at risk, pairwise comparisons showed doses to OARs as well as the integral dose were all statistically significantly lower in the proton plans compared to both X-ray techniques (all *p* < 0.003).

### 3.4. Impact of Breast Size

Correlations between breast size (represented by CTV in cm^3^) and doses to target volumes and organs at risk were tested using the non-parametric Kendall’s tau-B test with results shown in Table 4.

**Figure 2:**
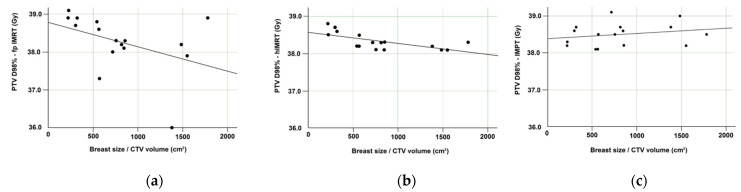
Correlation between breast size and PTV minimum dose (D98%) for: (**a**) fp-IMRT; (**b**) h-IMRT; (**c**) IMPT plans. All *n* = 16. Abbreviations: fp-IMRT—forward planned intensity-modulated radiation therapy; h-IMRT—hybrid intensity-modulated radiation therapy; IMPT—intensity modulated proton therapy.

Moderate negative correlations were seen between breast size and PTV coverage (D98%) for fp-IMRT and h-IMRT plans, indicating that larger breast size was associated with lower minimum PTV doses for the X-ray techniques as shown in Figure 2.

**Figure 3:**
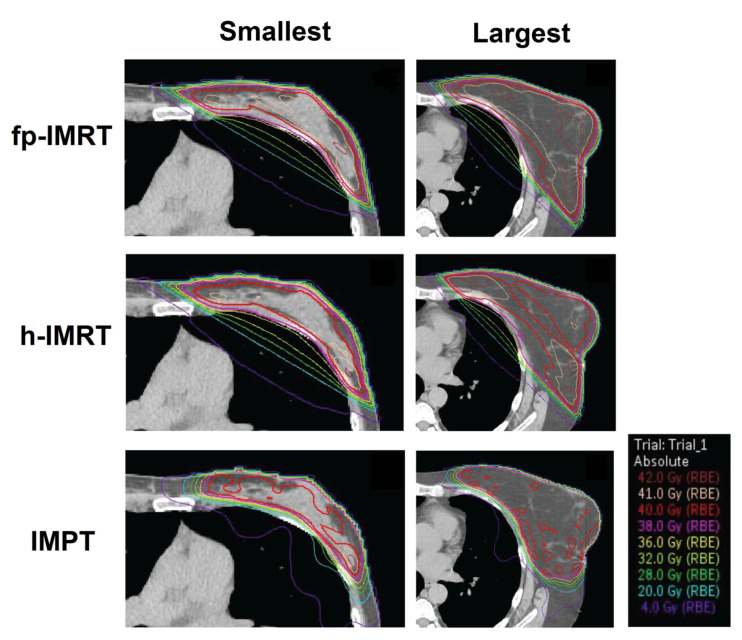
Dosimetry through the central transverse slice of each technique for two patients (smallest and largest breast size). Abbreviations: fp-IMRT—forward planned intensity-modulated radiation therapy; h-IMRT—hybrid intensity-modulated radiation therapy; IMPT—intensity modulated proton therapy.

A moderate positive correlation was observed between CTV volume and mean dose to the right lung for h-IMRT plans indicating larger breast size was associated with higher mean dose to the contralateral lung for this technique. No statistically significant correlations were evident for IMPT plans. Figure 3 shows dosimetry from the central transverse slice for each technique for the smallest and largest breast size.

## 4. Discussion

This is the largest retrospective planning study of early stage left-sided breast cancer patients in DIBH, comparing dosimetry between an IMPT technique using a single spot scanning beam, and two intensity modulated X-ray techniques in current clinical use. An additional investigation of relationships between breast size and resulting radiation dose was also undertaken. The key findings were that proton and X-ray techniques were equally effective in attaining acceptable target volume coverage, the h-IMRT and IMPT plans resulted in a significantly lower homogeneity index and significantly lower CTV and PTV mean and maximum dose compared to the fp-IMRT plan, and the CTV near minimum (D98%) was significantly lower for the h-IMRT plan compared to the IMPT. In terms of doses to organs at risk, the proton plans attained significantly lower radiation doses compared to both X-ray techniques to all organs at risk under investigation. Lastly, no correlation was seen between breast size and target volume coverage for the proton plans, however, in both X-ray techniques larger breast size was significantly associated with decreased PTV coverage.

Previous studies have shown that lower maximum doses and better homogeneity result in more favorable cosmetic outcomes for patients [21,22]. The h-IMRT and IMPT results were significantly different to the fp-IMRT in terms of lower plan maxima and better homogeneity. Mean radiation dose to the heart and LAD were significantly lower using IMPT compared to X-ray techniques, this was at least a 4-fold decrease in both LAD dose metrics or a 9-fold decrease for heart mean dose. Previous comparative studies investigating plans for whole breast only as well as whole breast with regional nodal irradiation (WB+RNI) have concluded that greater benefit of cardiac dose sparing is seen for WB+RNI patients [23,24]. Darby et al. [9] found that the risk of ischemic heart disease increases with mean heart dose by 7.4% per Gy, with no safe minimal dose threshold. This suggests that heart dose should be reduced as much as possible to limit this risk. As IMPT can reduce the heart dose to almost zero, it could be of particular benefit for breast cancer patients with known pre-existing heart conditions who are already likely to carry a higher risk of experiencing coronary events. Overall, our findings add further evidence for potential dose reduction with IMPT and further research such as the RadComp study will be able to determine whether dosimetric improvements translate to improvements in patient outcomes [10].

Mean lung dose and V18-20Gy are useful predictors for lung side effects. In this study, both the mean and V18Gy dose to the left lung showed a statistically significant reduction in proton therapy plans compared to X-ray plans. Taylor et al. [25] investigated long-term side effects to heart and lung following breast RT, and reported a 0.11 excess rate ratio per Gy for lung cancer meaning the patient’s underlying risk of lung cancer (due to smoking history, genetic predisposition, etc.,) was increased by 11% per Gy. While V18Gy is below tolerance levels for all techniques, the reduction in mean lung dose from almost 6 Gy in the X-ray plans to 1.4 Gy using protons may result in a clinically significant reduction of late side effects for these patients. Once again, as with heart dose, proton therapy for whole breast only may be indicated for patients with compromised lung function prior to treatment or those with an already high risk of lung cancer due to lifestyle and genetic factors.

Larger breast size has been shown to be an accurate predictor of adverse cosmetic outcome following conventional breast RT. This is due to dose heterogeneity, increase in high dose regions, and the bolus effect of skin creases in the inframammary and axillary regions [22,26]. The advent of 3D-planning and IMRT has improved dose heterogeneity for breast RT, however, poor cosmetic results for large-breasted patients are still observed [27,28]. The finding of a moderate negative correlation between CTV volume, which was representative of breast size, and target volume coverage for X-ray techniques, meant that as breast size increased, target coverage decreased. This finding is not surprising as both fp-IMRT and h-IMRT used opposed tangential beams to treat through the breast tissue. A large breast volume will generally result in a long path for the X-ray beam to travel through [29], where it becomes difficult to avoid high dose in the medial and lateral areas of the breast, and target volume coverage can be difficult to achieve. In contrast, the IMPT plans, with a single en-face beam and spots placed within the target volume to achieve the desired coverage, were not affected in this way by target volume size and this was confirmed with no correlations observed between CTV volume and target volume coverage for IMPT plans. To our knowledge this was the first study to evaluate associations between breast size and target coverage in proton therapy.

There were some limitations to this study. First, the relatively modest sample size was limited by data collection being confined to a single treatment center where the DIBH technique had been clinically implemented a short while before data collection. A larger, prospectively collected dataset would have enabled a more robust analysis. Second, due to uncertainties in skin dose calculation measured by treatment planning systems, skin dose was not taken into account in this study. A possible consideration for future studies would be to limit the dose to the 3–5 mm area between the skin and the PTV/CTV during plan optimization [30].

In conclusion, there were no significant differences in left breast DIBH target coverage between the X-ray and proton therapy techniques. Proton therapy was able to significantly reduce dose to all investigated organs at risk compared to the X-ray techniques. Breast size was shown to have a negative correlation with target coverage for X-ray plans only.

## Figures and Tables

**Table 1:** Patient characteristics, *n* = 16.

Characteristic	Mean (SD)
Age at diagnosis (years)	57.1 (11)
Breast size cm^3^	808 (494.2)
Diagnosis	*n* (%)
Invasive ductal carcinoma	10 (62.5%)
Invasive lobular carcinoma	3 (18.75%)
Papillary carcinoma	1 (6.25%)
Ductal carcinoma in situ	1 (6.25%)
Apocrine carcinoma	1 (6.25%)
Staging	
ypT0	1 (6.3%)
Tis	1 (6.3%)
T1	11 (68.7%)
T2	3 (18.7%)
N0	14 (87.5%)
N1mic	2 (12.5%)
M0	15 (93.8%)
Mx	1 (6.2%)
Grade	
1	4 (25%)
2	5 (31.3%)
3	5 (31.3%)
High	2 (12.5%)

**Table 2:** Differences in target volume parameters between fp-IMRT, h-IMRT, and IMPT plans, *n* = 16

**Plan Parameter**	fp-IMRT	h-IMRT	IMPT	Friedman’s	Wilcoxon’s
	Median (interquartile range)			*p*-value	*p*-value ^a^
CTV Dmean (Gy)	41.3 (41–41.4)	40.3 (40.2–40.4)	40.1 (40.1–40.2)	<0.001 *	
fp-IMRT vs. h-IMRT					<0.001 *
fp-IMRT vs. IMPT					<0.001 *
h-IMRT vs. IMPT					0.018
CTV D2% (Gy)	42.4 (42.1–42.5)	41.5 (41.3–41.7)	41.3 (41.1–41.4)	<0.001 *	
fp-IMRT vs. h-IMRT					<0.001 *
fp-IMRT vs. IMPT					<0.001 *
h-IMRT vs. IMPT					0.246
CTV D98% (Gy)	39.0 (38.5–39.4)	38.7 (38.6–39)	39.4 (39.2–39.5)	<0.001 *	
fp-IMRT vs. h-IMRT					0.066
fp-IMRT vs. IMPT					0.04
h-IMRT vs. IMPT					<0.001 *
PTV Dmean (Gy)	40.9 (40.8–41.2)	40.2 (40.1–40.3)	40.1 (40.1–40.1)	<0.001 *	
fp-IMRT vs. h-IMRT					<0.001 *
fp-IMRT vs. IMPT					<0.001 *
h-IMRT vs. IMPT					0.17
PTV D2% (Gy)	42.4 (42.1–42.5)	41.6 (41.3–41.8)	41.3 (41.1–41.5)	<0.001 *	
fp-IMRT vs. h-IMRT					<0.001 *
fp-IMRT vs. IMPT					<0.001 *
h-IMRT vs. IMPT					0.09
PTV D98% (Gy)	38.3 (38–38.9)	38.3 (38.1–38.5)	38.5 (38.2–38.7)	0.638	
V_38.05Gy_ (%)	98.8 (98–99.5)	98.7 (98.3–99.4)	98.6 (98.2–99)	0.829	
Homogeneity Index	0.1 (0.08–0.11)	0.08 (0.07–0.08)	0.07 (0.06–0.08)	<0.001 *	
fp-IMRT vs. h-IMRT					0.003 *
fp-IMRT vs. IMPT					<0.001 *
h-IMRT vs. IMPT					0.071

Abbreviations: CTV—clinical target volume; Dmean—mean dose received; Dx%—dose received by x% of structure; fp-IMRT—forward-planned IMRT; Gy—gray; h-IMRT—hybrid IMRT; IMPT—intensity modulated proton therapy; PTV—planning target volume; VxGy—volume of structure receiving xGy; ^a^ indicates Bonferroni correction at significance level *p* ≤ 0.017; * indicates statistically significant result.

**Table 3:** Differences in organ at risk parameters between fp-IMRT, h-IMRT, and IMPT plans, *n* = 16.

**Plan Parameter**	fp-IMRT	h-IMRT	IMPT	Friedman’s
	Median (interquartile range)			*p*-value
Heart				
Dmean (Gy)	0.8 (0.5–1.6)	0.9 (0.6–1.5)	0.1 (0.1–0.2)	< 0.001 *
D2% (Gy)	4.3 (2.8–15.6)	4.6 (3.3–14.5)	1.0 (0.6–1.7)	< 0.001 *
V5Gy (%)	1.5 (0.5–4.5)	1.8 (0.4–4.8)	0.2 (0.1–0.3)	0.009 *
V20Gy (%)	0.1 (0–1.6)	0.2 (0–1.3)	0 (0–0)	< 0.001 *
LAD				
Dmean (Gy)	3.7 (2.3–9.1)	3.6 (2–10.7)	0.6 (0.2–0.9)	< 0.001 *
D2% (Gy)	14.8 (4.7–34.5)	14.2 (4.2–35.7)	3.6 (0.9–5.4)	< 0.001 *
Lung_L				
Dmean (Gy)	5.9 (4.6–6.9)	5.6 (4.2–7.3)	1.5 (1.2–1.7)	< 0.001 *
V5Gy (%)	24.2 (19.1–28.3)	23.3 (18.1–29.9)	9.2 (7.9–10.1)	< 0.001 *
V18Gy (%)	12.9 (9.1–14.6)	12.5 (9–17.3)	1.4 (0.8–1.8)	< 0.001 *
Lung_R				
Dmean (Gy)	0.1 (0.1–0.1)	0.1 (0.1–0.2)	0 (0–0)	< 0.001 *
Breast_R				
Dmean (Gy)	0.2 (0.1–0.2)	0.2 (0.2–0.3)	0 (0–0.1)	< 0.001 *
Dmax (Gy)	9.0 (2.8–23.4)	10.1 (3.5–21)	2.5 (0.1–6.5)	< 0.001 *
ID_RVR_ (cm^3^.cGy)	31,129	30,699	16,830	< 0.001 *
	(18,880–42,414)	(19,050–43,383)	(12,265–22,766)	

Abbreviations: Dmean—mean dose received; Dx%—dose received by x% of structure; fp-IMRT—forward-planned IMRT; Gy—gray; h-IMRT—hybrid IMRT; ID—integral dose; IMPT—intensity modulated proton therapy; LAD—left anterior descending artery; RVR—remaining volume at risk; VxGy—volume of structure receiving xGy; * indicates statistically significant result.

**Table 4:** Correlation between breast size (CTV volume, cm^3^) and radiation dose to ROIs, *n* = 16.

**ROI**	Metric	fp-IMRT		h-IMRT		IMPT	
		*τ*_b_	*p*-Value	*τ*_b_	*p*-Value	*τ*_b_	*p*-Value
PTV	D98%	−0.434	0.021 *	−0.537	0.006 *	0.184	0.338
Heart	Dmean	0.269	0.158	0.288	0.124	−0.083	0.687
LAD	Dmean	0.034	0.857	0.017	0.928	−0.222	0.239
Lung_L	Dmean	0.317	0.087	0.259	0.162	−0.071	0.713
Lung_R	Dmean	0.345	0.112	0.519	0.017 *	n/r	n/r
Breast_R	Dmean	−0.308	0.126	−0.433	0.034	0.038	0.857

Abbreviations: Dmean—mean dose received; Dx%—dose received by x% of structure; fp-IMRT—forward-planned IMRT; h-IMRT—hybrid intensity-modulated radiotherapy; IMPT—intensity-modulated proton therapy; n/r—no result (no dose was recorded for Lung_R); PTV—planning target volume; ROIs—regions of interest; *τ*b—Kendall’s tau-B; * indicates statistically significant result.

## Data Availability

Restrictions apply to the availability of these data. Data was obtained from The Royal Adelaide Hospital, Adelaide, Australia and are available upon request from the authors with the permission of The Royal Adelaide Hospital.

## Appendix A

**Table A1:** List of regions of interest and anatomical definitions used in plan creation.

ROI Name	Definition	Reference
CTV	Includes apparent CT glandular breast tissue and lumpectomy CTV, incorporates consensus definition of anatomical borders per RTOG guideline	RTOG Breast Cancer Atlas [14]
PTV	CTV + 5 mm margin, limited to within Ext-5mm contour and excluding Lung_L contour	Cancer Institute NSW guidelines [15]
Breast_R	Right breast tissue as per CTV definition	RTOG Breast Cancer Atlas [14]
External	External contour of patient, extending at least 1 cm above and below PTV, including all OARs	
Ext-5mm	External contour, contracted 5 mm in anterior, posterior, superior, and inferior directions	
Heart	Entire heart as visible on CT	
LAD	Proximal 1/5th of the vessel, from the end of the left main coronary artery passing anteriorly behind the pulmonary artery. Mid 2/5th of the vessel descending anterolaterally in the anterior interventricular grooveDistal 2/5th of the vessel running in the interventricular groove and extending to the apex	A cardiac contouring atlas for radiotherapy [16]
Lung_L	Entire left lung as visible on CT	
Lung_R	Entire right lung as visible on CT	
RVR	External contour, avoiding the interior of PTV, Heart, LAD, Lung_L, Lung_R, and Breast_R contours	ICRU Report 83 [17]

Abbreviations: CT—computed tomography; CTV—clinical target volume; LAD—left anterior descending artery; PTV—planning target volume; ROI—region of interest; RTOG—Radiation Therapy Oncology Group; RVR—remaining volume at risk.

**Table A2:** Planning goals to the target and organs at risk for h-IMRT and IMPT plans.

Metric	Goal	Priority
PTV D_98%_	>38.05 Gy	Primary
PTV D_2%_	<42.8 Gy	
PTV D_mean_	40.05 Gy	
PTV HI	Close to zero	
Heart D_mean_	<3 Gy	Secondary
Heart V_21.5Gy_	<10%	
Lung_L V_18Gy_	<15%	
LAD	ALARA	
Lung_R	ALARA	
Breast_R	ALARA	

Abbreviations: ALARA—As Low As Reasonably Achievable; Dmean—mean dose received by structure; Dx%—dose received by x% of structure; Gy—gray; HI—homogeneity index, LAD—left anterior descending artery; OAR—organ at risk; PTV—planning target volume; VxGy—volume of structure receiving xGy.

**Table A3:** Summary of beam parameters.

Modality	Beam Angle, Median (Range)
X-ray plans	
Medial tangent (degrees)	308 (298–313)
Lateral tangent (degrees)	132 (121–138)
Proton plans	
Beam angle (degrees)	35 (32–38)
